# Old Wine in a New Bottle: Rebounding Vasectomy Practice in Low–Middle‐Income Country

**DOI:** 10.1002/puh2.70249

**Published:** 2026-04-24

**Authors:** Abraham Fessehaye Sium, Zerihun Beyene, Abrham Getachew, Malede Birara, Delayehu Bekele

**Affiliations:** ^1^ Department of Obstetrics and Gynecology St. Paul's Hospital Millennium Medical College (SPHMMC) Addis Ababa Ethiopia; ^2^ Department of Obstetrics and Gynecology King Faisal Hospital Rwanda, Africa Health Sciences University Kigali Rwanda; ^3^ Department of Public Health St. Paul's Hospital Millennium Medical College (SPHMMC) Addis Ababa Ethiopia

**Keywords:** contraception, low–middle‐income country, male contraception, male sterilization, no‐scalpel vasectomy (NSV), vasectomy

## Abstract

**Background:**

Despite existing overwhelming evidence on the effectiveness and safety of vasectomy as a permanent method of family planning, its use worldwide has been declining over the last 5 decades. Currently, there are several studies on knowledge and acceptance of vasectomy in Ethiopia from the client‐side (men). In this study, we investigated knowledge, attitude, and practice of vasectomy among family planning providers in Ethiopia.

**Methods:**

We conducted a descriptive survey study on knowledge, attitude, and practice of vasectomy among family planning providers in Ethiopia at St. Paul's Hospital Millennium Medical College over 2 months (from September 1, 2024, to October 30, 2024). Data were collected prospectively using a structured self‐administered questionnaire. Data were analyzed using SPSS version 23. Simple descriptive statistics were employed to analyze the data.

**Results:**

We approached 100 family planning providers, and 90 of them completed the response, making a response rate of 90%. Majority of the participants were obstetrics and gynecology resident physicians constituting 70% (63/90) followed by midwives who accounted for 13.3% (12/90). Seventy‐eight (86.7%) and 46 (51.1%) out of the 90 participants had good knowledge and favorable attitude towards vasectomy, respectively. Only 7 (7.8%) out of the total had good practice. The mean scores for knowledge, attitude, and practice were 80%, 50%, and 20%, respectively.

**Conclusion:**

Despite good knowledge and favorable attitude of family providers towards vasectomy in our study, significant gap was identified in the practice of vasectomy (less than 1 in 10 of the providers included in the study had good practice of vasectomy).

## Graphical abstract

The main objective of this study was to determine knowledge, attitude, and practice among family planning providers pertaining to vasectomy in Ethiopia. We found low level of practice (less than 1 in 10 of the participants had good practice) by respondents despite more than half of them having good knowledge and favorable attitude towards vasectomy. This implies that our current vasectomy program needs to focus on training family planning providers, parallel to expanding vasectomy services across Ethiopia.

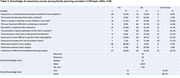

## 1 Introduction

Vasectomy is a commonly performed outpatient procedure for male contraception with high success rate, and various vasectomy techniques have been utilized, most notably the no‐scalpel vasectomy (NSV) [1]. Despite vasectomy's well‐recognized benefits, use of the method has plateaued globally and continues to languish in most low‐ and middle‐income countries, including Africa with 0.0 prevalence [2].

As a matter of fact, practice of vasectomy in Ethiopia remains very low despite high intention to use vasectomy as permanent method of contraception by married men in the country. A study done in the early 2000s in rural Southwest Ethiopia, which analyzed 200 men, documented high acceptance (79%) of vasectomy as a method of family planning [3]. Another similar study conducted in 2019 in Debre Tabor town (Northern Ethiopia), which included 402 participants, found the intention to use vasectomy was 19.6% [4]. Likewise, a 2018 community‐based cross‐sectional study conducted in the capital of the country, Addis Ababa, in which 422 married men participated, found 24% intention to use vasectomy as a method of family planning. The study further showed that 34.8% had good knowledge on vasectomy [5], which is comparable to 44.8% found in another similar study from Northwest Ethiopia that enrolled 827 male participants [6]. This fact about high acceptance rate of vasectomy in Ethiopia as method of contraception is further substantiated by findings of a recent (2022) study that found 18% intention to use vasectomy among married men from Addis Ababa city [7].

Although there is ample evidence in Ethiopia on knowledge and acceptance of vasectomy from the client‐side(men), there is no study that looked into knowledge, attitude, and practice of vasectomy by family planning providers (providers‐side). Our study presents the first evidence on knowledge, attitude, and practice of vasectomy among family planning providers at a family planning excellence center in Ethiopia.

## 2 Methods

### 2.1 Study Design, Study Setting, and Study Period

We conducted a descriptive survey study on knowledge, attitude, and practice of vasectomy by family planning providers at St. Paul's Hospital Millennium Medical College (SPHMMC) in Ethiopia, over 2 months from September 1 to October 30, 2024. SPHMMC is a leading medical college in Ethiopia with various specialty and sub‐specialty level care and trainings, including family planning fellowship program. The hospital is considered an excellence center for family planning in Ethiopia providing one of the most advanced and largest family services. Recently, we reintroduced a vasectomy program at this hospital in collaboration with St. Paul Institute for Reproductive Health and Rights (SPIRHR), an NGO affiliated with the hospital, with the purpose of expanding the scale and quality of vasectomy services across Ethiopia. As an initial step of implementing this program, we performed review of published papers on vasectomy in Ethiopia, and we identified that knowledge and acceptance of vasectomy by men in the country is well‐studied with favorable figures. However, there was no study that examined the same topic from the providers (family planning providers) perspective. To fill this research knowledge gap, we conducted the study. The main objective of our study was to determine family planning providers’ (nurses, midwives, and Ob‐Gyn resident physicians) knowledge, attitude, and practice of vasectomy at our center, SPHMMC.

### 2.2 Data Collection

Data were collected prospectively using a paper‐based self‐administered questionnaire which has four sections (socio‐demographic data, knowledge, attitude, and practice questions), and the questionnaire was adopted from previous similar study with modification of the items [8]. The inclusion criteria were healthcare providers assigned at the department of Obstetrics and Gynecology at SPHMMC, who are engaged in family planning service. We included physicians, nurses, midwives, and unit coordinators. The exclusion criteria were not volunteering to participate in the study.

### 2.3 Sample Size and Sampling Technique

No sample size calculation was employed. Convenience sampling technique was used to recruit participants—all respondents based on inclusion and exclusion criteria were included.

### 2.4 Data Analysis

Data were analyzed using SPSS version 23. Simple descriptive statistics were employed to analyze the data. We used scores of above 50% to define good knowledge, favorable attitude, and good practice. Although we used scores of 1–5 to assess the attitude component of the study based on Likert scale, the matrix for knowledge and practice scores was calculated as score of 3 points for correct answer, score of 0 points for completely missing the correct answer, and score of 1 for a neutral response.

## 3 Results

We approached 100 family planning providers, and 90 of them completed the response, making a response rate of 90%. Two‐thirds (67.8%, 61/90) of the respondents were male respondents (Table 1). Majority of the participants were Ob‐Gyn resident physicians, constituting 70% (63/90) followed by midwives who accounted for 13.3% (12/90).

**TABLE 1:** Socio‐demographic characteristics of family planning providers, in Ethiopia, 2024, *n* = 90.

Variable	Category	*n*	%
Sex	Male	61	67.8
	Female	29	32.2
Age (years)	Mean	30.6 (±3.6)	
Profession	Ob‐Gyn faculty/fellow	10	11.1
	Ob Gyn resident	63	70.0
	Midwife	12	13.3
	Nurse	3	3.3
	Other	2	2.2
Place of work	SPHMMC	84	93.3
	Outside SPHMMC	6	6.7
Years of experience (years)	Median	4 (1–23)	
Marital status	Unmarried	44	49.9
	Married	46	51.1
Religion	Orthodox	55	61.1
	Muslim	15	16.7
	Protestant	16	17.8
	Catholic	3	3.3
	Other	1	1.1

Abbreviation: SPHMMC, St. Paul's Hospital Millennium Medical College.

Seventy‐eight (86.7%) and 46 (51.1%) out of the 90 participants had good knowledge and favorable attitude towards vasectomy, respectively (Table 2). Only seven (7.8%) out of the total had good practice. Their mean scores for knowledge, attitude, and practice were 80%, 50%, and 20%, respectively.

**TABLE 2:** Knowledge of, attitude towards, and practice of vasectomy scores among family planning providers in Ethiopia, 2024, *n* = 90.

KAP component	Category	*n*	%
Knowledge	Poor	12	13.3
	Good	78	86.7
	Mean score	80	
Attitude	Unfavorable	44	48.9
	Favorable	46	51.1
	Mean score	50	
Practice	Poor	83	92.2
	Good	7	7.8
	Mean score	20	

Although the mean scores of knowledge and attitude were good, the scores for some important individual knowledge and attitude questions were low. For example, a quarter (25.6%, 23/90) of the participants responded incorrectly to whether vasectomy impairs ejaculation, and another more than half (62.2%, 56/90) of them had “I don't know response” (Table 3). Similarly, more than a quarter (28.9%, 26/90) agreed that they consider tubal ligation a more appropriate permanent method over vasectomy in Ethiopian setting (Table 4). Likewise, in the practice component, more than 90% of the participants reported that they have never assisted or seen vasectomy performed (Table 5).

**TABLE 3:** Knowledge of vasectomy scores among family planning providers in Ethiopia, 2024, *n* = 90.

Variable		Yes		No		Don't know	
		*n*	%	*n*	%	*n*	%
Vasectomy is considered best permanent method of contraception?		68	75.6	14	15.6	8	8.9
The normal functioning of the testis is altered?		8	8.9	75	83.3	7	7.8
There is usually a reduction or loss of libido in the male?		10	11.1	67	74.4	13	14.4
The man may have difficulty in achieving an erection?		8	8.9	69	76.7	13	14.4
Ejaculation is impaired after vasectomy		23	25.6	56	62.2	11	12.2
The procedure requires admission of the client routinely?		7	7.8	68	75.6	15	16.7
The procedure is best done under general anesthesia?		8	8.9	67	74.4	15	16.7
Vasectomy is more difficult to perform than tubal ligation		9	10.0	60	66.7	21	23.3
Vasectomy increases the risk of prostate cancer?		1	1.1	70	77.8	19	21.1
Vasectomy requires minor surgery		80	88.9	4	4.4	6	6.7
Vasectomy requires major surgery		4	4.4	79	87.8	7	7.8
Vasectomy is effective immediately following surgery		17	18.9	58	64.4	15	16.7
Overall knowledge score	Minimum	10					
	Maximum	36					
	Median	31					
	Mean	28.83					
	Std. dev	7.35					
Overall knowledge level	Poor	12				13.3%	
	Good	78				86.7%	

**TABLE 4:** Attitude towards vasectomy among family planning providers in Ethiopia, 2024, *n* = 90.

Variable	Strongly disagree		Disagree		Neither agree or disagree		Agree		Strongly agree	
	*n*	%	*n*	%	*n*	%	*n*	%	*n*	%
Vasectomy is not an appropriate family planning option in Ethiopia because it decreases the man feeling of being a man	30	33.3	35	38.9	14	15.6	10	11.1	1	1.1
It should not be considered because it curbs the man's ability to marry another wife or have another partner if divorce happens to him	25	27.8	21	23.3	20	22.2	19	21.1	5	5.6
I am convinced the average Ethiopian male will not accept vasectomy even when a most appropriate option	8	8.9	8	8.9	13	14.4	48	53.3	13	14.4
I will opt or consider vasectomy when I have desired family size/completed family sized (for male respondent)	14	23.0	15	24.6	13	21.3	15	24.6	4	6.6
I will recommend or consider vasectomy to my partner than having tubal ligation (for female respondent)	3	10.3	4	13.8	9	31.0	5	17.2	8	27.6
I recommend vasectomy to my relative/close friends as one of safe permanent FP method	7	7.8	13	14.4	19	21.1	39	43.3	12	13.3
I consider tubal ligation a more appropriate option for permanent contraception in our setting than vasectomy	16	17.8	18	20.0	25	27.8	26	28.9	5	5.6

**TABLE 5:** Vasectomy practice by family planning providers practice of vasectomy in Ethiopia, 2024, *n* = 90.

Variable	Category	*n*	%
Have you received training on family planning, including counseling?	Yes	52	57.8
	No	38	42.2
Have you received training on permanent methods of family planning including vasectomy?	Yes	21	23.3
	No	69	76.7
Have you ever assisted or done vasectomy before?	Yes	6	6.7
	No	83	92.2
	Don't know	1	1.1
Have you ever seen a vasectomy being done?	Yes	7	7.8
	No	82	91.1
	Don't know	1	1.1
Do you have any relative or friend for whom vasectomy has been done?	Yes	3	3.3
	No	86	95.6
	Don't know	1	1.1

## 4 Discussion

The main objective of our study was to determine knowledge, attitude, and practice levels of family planning providers pertaining to vasectomy as a permanent contraception method. We found low level of practice (less than 1 in 10 of the participants had good practice) by respondents despite more than half of them having good knowledge and favorable attitude towards vasectomy.

Current evidence indicates that vasectomy use is plagued by low demand pointing towards making significant efforts towards robust demand generation [9]. However, what is equally important is drawing on broader social and behavioral change of communication efforts (including among healthcare providers–family planning providers) aimed at increasing practice of vasectomy (service expansion)—well‐trained, skilled, and motivated staff is one of the main pre‐requests for successful provision of high‐quality vasectomy services [10]. To this end, there are few studies that divulge knowledge, attitude, and practice on vasectomy among family planning providers in low‐income countries. A recent 2020 study from Rwanda in which 290 healthcare providers (nurses, doctors, and midwives) were surveyed revealed an overall good knowledge and attitude towards vasectomy—58.3% and 69.7% of participants had good knowledge and positive attitude, respectively [8]. Another cross‐sectional study from Malaysia conducted in 2019 that explored attitude towards vasectomy and its acceptance as a method of contraception among clinical‐year medical students found an overall 60.9% of participants had a positive attitude towards vasectomy and 76.0% showed good acceptance [11]. A recent study conducted in India that aimed at estimating the knowledge of gynecologists, their attitude, and prevalence of practice of NSV concluded that knowledge of gynecologists about NSV was inadequate with low level of practice (out of 447 gynecologists, only 14/3.1% were trained in performing NSV) [12]. Although 86.7% (78/90) and 51.1% (46/90) of the study participants in our study had good knowledge and favorable attitude towards vasectomy, their practice level was very low (only 7.8% or 7/90 had good practice) with a mean score of practice of 20%. These findings are consistent with findings of previous studies (discussed above) and hint a significant amount of work remains to be done to up‐scale the practice of vasectomy by family planning providers. The focus should be on the training aspect, which is key to achieving the second vital need to securing successful vasectomy program next to demand generation.

Although our study presents these interesting results, it has some limitations. Lack of proper sample size allocation and sampling technique are the main limitations. Not including qualitative data (which could have helped to assess factors influencing the very low level of practice) and being a single‐center study are the other limitations.

## 5 Conclusion

Though knowledge and attitude of family providers towards vasectomy were found to be good in our study, significant gap was identified in the practice component. This implies that our current vasectomy program needs to focus on training family planning providers, as we gear up our efforts towards rebranding the old wine in new bottle—better vasectomy counseling and practice.

## Author Contributions

Delayehu Bekele, Zerihun Beyene, Abrham Getachew, and Abraham Fessehaye Sium contributed to the conception and development of the study design, data collection, and data analysis. Abraham Fessehaye Sium, Malede Birara, and Delayehu Bekele performed data interpretation and manuscript write‐up. The final manuscript was edited by Abraham Fessehaye Sium. All authors critically revised the article for intellectual content. All authors reviewed the final manuscript and approved its submission for publication.

## Funding

This study received funding from St. Paul Institute for Reproductive Health and Rights (SPI‐RHR).

## Ethics Statement

Formal ethical clearance letter was obtained from institutional review board of St. Paul Hospital Millennium Medical College.

## Consent

Written informed consent was obtained from study participants.

## Conflicts of Interest

The authors declare no conflicts of interest.

## Data Availability

Data are available on request from authors.
